# Screening for parent and child ADHD in urban pediatric primary care: pilot implementation and stakeholder perspectives

**DOI:** 10.1186/s12887-023-04082-2

**Published:** 2023-07-13

**Authors:** Joyce H. L. Lui, Christina M. Danko, Tricia Triece, Ian M. Bennett, Donna Marschall, Nicole E. Lorenzo, Mark A. Stein, Andrea Chronis-Tuscano

**Affiliations:** 1 Department of Psychology, University of Maryland, College Park, MD USA; 2 Department of Psychology, Concordia University, 7141 Sherbrooke West, PY-146, Montreal, QC Canada; 3 Family Medicine and Psychiatry & Behavioral Sciences, University of Washington, Seattle, WA USA; 4 Whole Bear Care, Children’s National Hospital, Washington, DC USA; 5 Department of Psychology, American University, Washington, DC USA; 6 Psychiatry and Behavioral Medicine, Seattle Children’s Hospital, Seattle, WA USA

**Keywords:** ADHD, Screening, Parent mental health, Low-income, Pediatric primary care

## Abstract

**Background:**

ADHD commonly co-occurs in children and parents. When ADHD is untreated in parents, it contributes to negative child developmental and treatment outcomes. Screening for parent and child ADHD co-occurrence in pediatric primary care may be an effective strategy for early identification and treatment. There is no data on whether this screening model can be implemented successfully and there exists limited guidance on how to effectively approach parents about their own ADHD in pediatric settings. Even greater sensitivity may be required when engaging with families living in urban, low SES communities due to systemic inequities, mistrust, and stigma.

**Methods:**

The current pilot study described the first 6 months of implementation of a parent and child ADHD screening protocol in urban pediatric primary care clinics serving a large population of families insured through Medicaid. Parents and children were screened for ADHD symptoms at annual well-child visits in pediatric primary care clinics as part of standard behavioral health screening. Independent stakeholder group meetings were held to gather feedback on factors influencing the implementation of the screening and treatment strategies. Mixed methods were used to examine initial screening completion rates and stakeholder perspectives (i.e., parents, primary care office staff, pediatricians, and behavioral health providers) on challenges of implementing the screening protocol within urban pediatric primary care.

**Results:**

Screening completion rates were low (19.28%) during the initial 6-month implementation period. Thematic analysis of stakeholder meetings provided elaboration on the low screening completion rates. Identified themes included: 1) divergence between provider enthusiasm and parent hesitation; 2) parent preference versus logistic reality of providers; 3) centering the experiences of people with marginalized identities; and 4) sensitivity when discussing parent mental health and medication.

**Conclusions:**

Findings highlight the importance of developing flexible approaches to screening parent and child ADHD in urban pediatric health settings and emphasize the importance of cultural sensitivity when working with marginalized and under-resourced families.

**Trial registration:**

NCT04240756 (27/01/2020).

**Supplementary Information:**

The online version contains supplementary material available at 10.1186/s12887-023-04082-2.

## Background 

Attention-Deficit/Hyperactivity Disorder (ADHD) is a common disorder that affects both children and adults. Given the high heritability of ADHD [1], multiplex families, where both a parent and child have ADHD, is a common occurrence. Estimates suggest 25–50% of children with ADHD have at least one parent who also has ADHD [2]. Parent and child ADHD have transactional effects, whereby parent ADHD symptoms and behaviors impact the trajectory and outcomes of child ADHD and child ADHD symptoms impact parent’s functioning and parenting behaviors [3]. Parent ADHD can also significantly impact treatment outcomes for child ADHD. Specifically, parents with ADHD symptoms are likely to struggle with organization and consistency, which are skills necessary for effective management of child medication and are integral to behavioral treatments for child ADHD [4, 5]. Indeed, Friedman et al. [6] found that parent ADHD symptoms were negatively associated with the practice of behavioral parent training strategies between sessions. In addition, children in multiplex ADHD families experience more peer difficulties, have more severe ADHD symptoms and comorbidities, and demonstrate diminished improvement in response to behavioral treatment relative to children with ADHD whose parents do not have ADHD [4, 5]. These findings highlight the importance of identifying and treating ADHD simultaneously in both parent and child early.

Disparities in diagnosis and treatment related to socioeconomic status (SES) exist for individuals with ADHD. Primary care providers working in under-resourced urban settings with higher poverty rates report more challenges in conducting appropriate diagnostic assessments for ADHD compared to those in suburban settings [7], which may be in part due to higher psychosocial stress in families and higher rates of trauma exposure and related symptoms among children in under-resourced settings, adding complexity to differential diagnoses [8]. Furthermore, overall rates of psychosocial intervention use for ADHD remain low [9], in part due to barriers such as misconceptions and stigma associated with ADHD and mistrust towards health professionals [10]. A lack of insurance coverage has also been associated with decreased access to ADHD care [11]. Research also documents poorer quality of care among racial and ethnic minoritized youth [12–14], which is often confounded by SES. For example, Black and racially minoritized families are more likely to be living in poverty than their White counterparts [15].

Implementation of ADHD interventions in community and primary care settings face several barriers that may be distinct from private, suburban clinics, including limited primary care provider education on ADHD, lack of resources and time involved in diagnosing and treating a complex disorder such as ADHD, and the need to coordinate with others (e.g., parents, schools, other health professionals) to manage the disorder [16]. Families from under-resourced backgrounds also experience logistical barriers to accessing and engaging with mental health care such as long wait lists, difficulties with scheduling during traditional work hours, and traveling to the visit [17]. These barriers are often exacerbated by the daily stressors and demands that are associated with living in poverty or under-resourced neighborhoods, which keep families from seeking mental health supports. A recent study found that children living in lower-resourced neighborhoods (e.g., low on domains of income, employment, education, health, living environment, and barriers to housing/services, and high on crime) were more likely to receive medication for ADHD [18]. The authors speculated that families with limited resources may be less likely to access and/or have less access to first-line evidence-based psychosocial interventions, thus increasing their likelihood of receiving medication to treat ADHD [18]. Alternatively, families may lack financial means to participate in supports that may reduce child ADHD impairment (e.g., sports or other extracurricular activities, educational support) and corresponding need for medication [18]. The robust associations between SES and ADHD diagnosis and treatment suggest additional considerations when working with families from low SES backgrounds.

Pediatric primary care represents an opportunity for the early identification of multiplex ADHD, given that the American Academy of Pediatrics (AAP) recommends routine evaluation and treatment of ADHD in children beginning at age 4 [19]. Despite the prevalence of ADHD in parents and potential impact on the child, it is not standard practice to screen for parent ADHD in pediatric primary care. However, the AAP recommends screening for postpartum depression for new mothers during well-child visits [20]. The precedent of screening for postpartum depression in pediatric primary care may thus serve as a model for the screening of other parent mental health concerns. Parallel to findings concerning the impact of parent ADHD on child development and outcomes, parental depression has been found to impact child’s cognitive development, language development, mother–child bonding and attachment, risk of child behavioral difficulties, and lower academic achievement [21, 22]. Chaudron et al. [23] found that implementing universal screening for postpartum depression in a large pediatric practice resulted in a significant increase in the detection of maternal postpartum depression. Additionally, Leung et al. [24] found that mothers who were screened for postpartum depression and received treatment had better mental health outcomes at 6 months after their child’s birth relative to mothers who received standard clinical care (i.e., no universal screening for postpartum depression). These findings illustrate that screening for parent mental health in pediatric primary care settings can be feasible and beneficial to families.

No study, to date, has examined screening for parent and child ADHD in pediatric primary care. Although screening for parent mental health in pediatric primary care has benefits, there are challenges to implementing screening protocols in this setting. For example, parents may not feel comfortable discussing their own mental health symptoms with their child’s provider due to lack of trust or worries about being judged [25]. Provider time constraints, variability in the quality and extent of providers’ mental health training, inconsistent and limited reimbursement for mental health screening, stigma around mental health, and concerns about patient privacy and literacy have also been identified as barriers to the implementation of universal screening in primary care [26]. Additionally, families from ethnic minoritized groups, lower SES backgrounds, who are non-native English speaking, or who are uninsured or publicly insured are screened at a lower rate than other demographic groups [27–29]. The disparity is evident for both child [30] and parent mental health screening [31]. In a systematic review of postpartum depression screening for low-income women, Hansotte et al. [32] found several barriers, including parental fear that their child would be taken from them due to their mental health, perceptions that “good mothers” do not have depression, and previous negative experiences with mental health services. Similar barriers and challenges are likely to exist when screening for parent ADHD in under-resourced communities. These challenges related to systemic, provider, and patient characteristics are important to consider when attempting to implement universal screening for parent and child ADHD in pediatric primary care.

At present, there is little guidance on how to effectively approach parents about their own ADHD in a pediatric care setting, and broaching this topic with under-resourced families may require even greater sensitivity due to systemic inequities, mistrust, and stigma. The current pilot study describes initial efforts of implementing a protocol to screen for parent and child ADHD in urban pediatric primary care clinics and stakeholder perspectives on challenges and strategies to effectively engage with families living in under-resourced communities around topics of parent mental health, ADHD screening, and treatment of ADHD in multiplex families. The study period spanned from August to November 2020, during the COVID-19 pandemic and the Black Lives Matter movement following the police murder of George Floyd in May 2020.

## Methods

### Study context

The current pilot study is part of a larger hybrid effectiveness-implementation randomized controlled trial (RCT; NCT04240756; 27/01/2020) examining the effectiveness and implementation of two treatment strategies for families with ADHD. The RCT is conducted in partnership with Children’s National Hospital (CNH), a Mid-Atlantic urban pediatric hospital in the U.S. that serves a large population of low-income children and families in the metropolitan Washington, DC area. Table 1 displays demographic data from the DC Health Matters Collaborative (https://www.dchealthmatters.org) comparing the service areas of the primary care clinics to all of DC. In the U.S., the AAP recommends well-child visits at regular intervals, which are regular exams for children beginning from age 0 to 21. Visits allow for comprehensive assessments and focus on the child’s development. Parents and children were screened for elevated ADHD symptoms at annual well-child visits in pediatric primary care clinics via questionnaires as part of standard universal behavioral health screening. The Strengths and Difficulties Questionnaire (SDQ) [33] was already used in clinics to screen for child ADHD symptoms and the Adult ADHD Self-Report Scale for DSM-5 (ASRS) [34] was added to screen for parent ADHD. Data from the screening packet is subsequently entered into children’s medical chart or collected by the research team. A score ≥ 7 on the Hyperactivity subscale on the SDQ and a score ≥ 14 on the ASRS indicated a positive screen. All procedures (for both the RCT and stakeholder meetings) were performed in accordance with the Declarations of Helsinki guidelines and were approved by institutional review boards at Seattle Children’s Hospital and Children’s National Hospital.

**Table 1:** Demographics for DC and DC Wards

	Overall DC	Southeast DC (Ward 7)	Southeast DC (Ward 8)	Northwest DC (Ward 5)
Race				
AAPI	4.58%	.40%	.50%	3.51%
African American/Black	43.46%	91.49%	91.61%	54.53%
American Indian/Alaskan Native	.36%	.31%	.25%	.44%
White	42.72%	3.19%	4.38%	31.93%
Another race	5.29%	2.01%	.81%	5.73%
Multiracial	3.59%	2.61%	2.43%	3.87%
Median Household Income	$102,806	$50,130	$44,665	$104,296^a^
% Families with Children Living Below Poverty Threshold	7.57%	15.16%	18.59%	4.65%
Highest Attained Education				
Less than 9^th^ Grade	3.43%	3.76%	3.26%	3.78%
Some High School or Below	4.95%	9.86%	9.58%	5.15%
High School	16.17%	38.82%	37.53%	17.03%
Some College	12.82%	22.80%	25.68%	14.95%
Associate/Bachelor’s	28.67%	16.14%	15.53%	30.58%
Master’s or Higher	33.97%	8.64%	8.41%	28.51%
Unemployment	6.53%	14.76%	16.02%	5.82%
Population	692,263	77,456	77,756	86,794

The statistics represent March 2022 demographics extracted from DC Health Matters Collaborative (https://www.dchealthmatters.org)

*AAPI* Asian American/Pacific Islander

^a^Significant variability in median household income exists among the two most populous race/ethnicities in Ward 5: $69,873 among African Americans/Black residents and $179,284 among White residents

### Stakeholder participants & meetings

*Stakeholder Participants.* Participants were recruited from a pediatric primary care/hospital-connected network of clinics using a purposive sampling approach. Stakeholders were “individuals who play a role in or are otherwise impacted by the implementation effort” [35] and included parents with ADHD or parents of a child with ADHD, pediatricians, behavioral health providers, or office staff at the primary care clinics. All participants provided informed consent specific to stakeholder participation and were compensated $50 per meeting. Nine stakeholders were included in the current pilot study: 2 parents, 2 pediatricians, 2 office staff, and 3 behavioral health providers (33% Black; 33% White; 22% Asian; 11% other). All pediatricians, behavioral health providers, and office staff worked at the urban primary care clinics. Both parents had children who receive care at the urban primary care clinics. Seven stakeholders were recruited for the first stakeholder meeting, and two additional stakeholders, one pediatrician and one primary care office staff, joined the group prior to the second meeting. The sample was predominantly female (89%). The mean age of participants was 35.33 years (range 31–49 years). 

*Stakeholder Meetings*. The stakeholder group met two to three times a year throughout the duration of the RCT, with the explicit purpose of providing feedback on the implementation of the RCT including the screening workflow and the multiplex ADHD treatment model. Meetings were moderated by study investigators (ACT and IB) with observation by note takers. All stakeholder meetings occurred via telehealth due to the COVID-19 pandemic and were recorded via Zoom. Audio recordings of the two stakeholder meetings were transcribed verbatim by research assistants and analyzed in NVivo 12 [36]. For the current study, meetings were held in June and November of 2020 and lasted approximately 90 min. Semi-structured interview guides were developed by the members of the investigative team (JL, CD, ACT, and IB) and were guided by the Consolidated Framework for Implementation Research (CFIR) [37]. Questions focused on factors that might influence the implementation of screening for parent and child ADHD in pediatric primary care and strategies for treating parent and child ADHD. Discussions involved feedback on screening rate and workflow and the treatment model as well as recruitment and engagement strategies. Example prompts included*:*

- How does the parent ADHD screening questionnaire fit with clinic workflow?
- How can we increase ADHD screening at the clinics?
- What are your thoughts about our recruitment approaches?
- What is the best way to talk with parents about medication and address stigma around medication?

### Analytic Plan

A mixed methods approach was used- quantitative data to examine screening completion rates and qualitative data to expand on the screening rate observed and to elaborate on the screening implementation context. To describe screening implementation, rates of screening completion and positive and negative screen rates were calculated. Thematic analysis was conducted using Braun and Clarke’s procedure [38] to identify and analyze patterns in the stakeholder meeting transcripts. Thematic analysis was chosen because it is not theoretically bounded and allowed for both an inductive and deductive approach to analysis. We used the CFIR framework to guide a priori codes, but also allowed the possibility of new emergent codes. Coders first reviewed the two meeting transcripts in full. Through an iterative process, a codebook was developed and applied to the transcripts, with 100% of the transcripts double-coded for reliability. To identify, review, and define themes, the coding team and authors engaged in iterative collaborative discussions.

## Results

Across a 6-month period from June to November, 1,105 children aged 3–8 years old attended a well-child visit at one of the primary care clinics. A total of 202 (18.28%) screening forms were returned. Examination of returned forms indicated that the majority of parents (59.41%) opted out of completing the adult ADHD screener. Among the 82 families who returned and completed the screening packet, 17 (20.73%) families screened positive (i.e., both parent and child had elevated ADHD symptoms). Another 14.6% of families had either parent (7.3%) or child (7.3%) screen positive for elevated ADHD symptoms (but not both), and 50% of families had no elevated symptoms in either parent or child. Overall, initial efforts at implementing a protocol to screen for parent and child ADHD in pediatric primary care yielded low screening completion rates. However, the positive screening rate suggests encounters with multiplex ADHD families in primary care may be common.

Stakeholder feedback provided context and helped further our understanding of challenges and strategies to overcome barriers to screen and treat multiplex ADHD families in primary care. Thematic analysis identified the following themes: 1) divergence between provider enthusiasm and parent hesitation; 2) parent preference versus logistic reality of providers; 3) centering the experiences of people with marginalized identities; and 4) sensitivity when discussing parent mental health and medication. These themes are discussed below and excerpts are included for illustrative purposes.

### Theme 1: divergence between provider enthusiasm and parent hesitation

Although pediatricians and behavioral health providers voiced benefits of incorporating parent and child mental healthcare in a singular, integrated treatment approach, parents expressed hesitation about this approach. Notably, providers reported advantages of focusing on the family unit over current models of care that focus strictly on the child in primary care, such as being able to help parents in-house rather than relying on referrals to community resources, which are scant and varied in quality.

*“So, the fact that this study kind of has figured out a way to integrate both child and parent mental health in one treatment package is really nice and I think that's novel in a lot of ways. It’s always a hard balance for us like who can we send out into the community and who we can keep, just because of a lack of community resources. So, if this helps us keep more families and be able to provide the treatment within the model integrated in primary care, I think that’s wonderful.”*—Pediatrician

Providers also recognized the transactional nature of parent and child symptoms, such that child symptoms impact parents and vice versa, and that addressing parent mental health can ultimately improve children’s treatment and outcomes. Overall, providers highlighted benefits of treating the family holistically. Although providers were enthusiastic about an integrated care model, parents expressed hesitation about addressing parent mental health within the context of a well-child visit in pediatric primary care. Specifically, parents may be hesitant to take focus away from their child during well-child visits, even though parent ADHD significantly impacts child outcomes. In particular, parents perceived that discussing parent mental health would take away time dedicated for their children.

“*But from a parent’s point of view, they give me a questionnaire about my mental history, I would not want to do that cause that’s opening up a whole another door that I don’t think would be appropriate at the time, like I’m trying to get the best care for my son, because like my issues shouldn’t be his issue.”*—Parent

Parents may also not expect questions about their own mental health in pediatric primary care and these questions may feel “invasive” and elicit unpleasant memories, distress, or perceptions of blame for their child’s difficulties.

*“Like me being a parent and going to the doctor, if someone was to ask me to fill out a questionnaire about my mental health and my son, I would be intimidated and I don’t think I would quite share because I’m here for the child, and with me depending on the personal circumstance situation, that’s a lot to pull from.”*—Parent

### Theme 2: parent preference versus logistic reality of providers

Another theme relates to tension between parent preference for having conversations about parent mental health with providers and providers having limited time to engage in these conversations due to their full caseloads. Parents expressed low acceptance identifying and addressing their own ADHD via screening questionnaires and providers also noted challenges of utilizing this strategy for screening for parent mental health. Parents described the risk that screening for parent mental health via questionnaires might trigger painful experiences. Furthermore, parents reported experience with “screening fatigue,” where the volume of paperwork at well-child visits was overwhelming for many parents and suggested that adding extra forms to screen for parent mental health is not preferred.

*“A lot of people don’t pay attention to the paperwork. I know I go to the doctor, I still gotta fill out postpartum form for my daughter that I don’t wanna do but I have to do it, so I couldn’t even imagine filling out a postpartum form then a whole other form about my mental health, or what I've been through and then my child. Like it’s a lot. “*—Parent

Providers reported similar observations of screening fatigue among families that they serve, particularly for families with young children due to the large number of health and developmental screening recommended by the AAP.

*“Just from our own experience at our clinic is that parents, especially this age group, they get to the clinic and they have these Bright Futures handouts to complete and everything else, and then they do the SDQ with all the behavioral questions and I think by the time they get to that page, they’re just like, you know […] ‘I don’t wanna do any of this stuff.’”*—Pediatrician

Instead of completing screening questionnaires, participants expressed favorable attitudes towards having conversations with trusted providers to engage parents about their mental health. Providers’ established relationship with parents made them well-equipped to have these sensitive conversations. Additionally, parents and providers described the benefits of considered and careful conversations with a trusted provider for introducing new treatment approaches, such as integrated care.

*“The doctor and the parent have a good relationship. I don’t think if someone else told me about it, I know I probably wouldn’t have done it, ‘cause I don’t know you, I don’t trust you.”*—Parent

However, engaging parents in sensitive conversations with trusted providers require time, which providers may not have. Although pediatricians, behavioral health providers, and parents repeatedly noted the importance of these conversations, the time investment for carrying out such deliberate conversations seemed at times incompatible with pediatricians’ workflow (e.g., managing back-to-back appointments, completing referrals) during typical well-child visits. Faced with these competing demands on time, pediatricians acknowledged the challenges of taking such an approach.

*“'Cause just to give you an idea, on the schedule, we could be scheduled for, you know, up to 21 patients a day on a typical well-visit schedule. So you're seeing a lot of physicals back-to-back and you really don't have that much wiggle room for kind of these more delicate conversations for a study that could be extremely helpful.”*—Pediatrician

### Theme 3: centering the experiences of people with marginalized identities

Another major theme was the importance of centering mental health treatment around the historical and current negative experiences faced by many marginalized communities as a whole and as individuals. Explicitly eliciting, attending, and listening to the experiences of individuals with marginalized identities was viewed as critical. For example, providers noted the need for sensitivity to the historical mistreatment of Black people by medical professionals, especially in light of the Black Lives Matter movement and elevated focus on the patterns of systemic racial discrimination and racially motivated violence experienced by Black individuals across the country.

*“I think that, it is something that has been a part of our work if you work with Black and African American families that this has always been part of our treatment and our sensitivity but is definitely heightened right now. I think yes, when talking about medication we have to recognize the immense history of mistreatment in the medical field towards Black and African American patients.”*—Behavioral Health Provider

Parents and providers emphasized discrimination faced by Black boys with ADHD. Parents expressed concerns about the overdiagnosis of ADHD in Black boys, as well as fears about how others perceive or react to their behaviors. The reality of the negative consequences (e.g., racially motivated discrimination) disproportionately experienced by Black children was cited as a major source of concern:

*“I see so many parents and mothers specifically, but also fathers of kids with ADHD, who their fears about their kid’s behavioral challenges and their impulsivity is so much more heightened because they know what the reality is that Black boys and Black children face in the world.”*—Pediatrician

*“My son being Black, you know, a lot of Black kids have been diagnosed with ADHD. So that’s just tough on its own, you know, being a mom and having a Black son and oh, he has ADHD, so basically just trying to change and break the barrier and supporting my son …"*—Parent

### Theme 4: Sensitivity when discussing parent mental health and medication

This theme centered around the sensitivity required when speaking with parents about their mental health as well as treating mental health with medications due to stigma. Significant stigma related to mental health and seeking help from a psychologist or psychiatrist remains prevalent, especially among underserved and marginalized communities.

*“When it comes to mental health in the Black community, it’s something that people don’t like to discuss because that can trigger to other things.”*—Parent

Parents and providers described various strategies for effectively reaching parents who may be facing these hesitations. They recommended using straight forward language and avoiding clinical labels for parents who may have been previously misdiagnosed or undiagnosed. Specifically, when asking about ADHD symptoms, parents and providers shared that it may be more effective to describe symptoms of ADHD rather than use the term “ADHD”:

*“'Cause a lot of people may have felt difficulty focusing or juggling responsibilities and things like that. A lot of people could relate to that. Versus not as many people could definitely say, "Oh, I think I have ADHD and so does my child."*—Pediatrician

Sensitivity is also required when addressing medication for treating ADHD, exemplified by statements such as “I don’t believe in medication.” Parents’ unfavorable attitudes towards being medicated or having their child medicated were echoed by providers in their experiences working with families in an urban primary care setting.

*“With the stigma against medication because like [Parent]’s mentioning, majority of parents that we see are still hesitant about medication for their own child versus even for themselves because they’ve had such poor experience with mental health.”*—Behavioral Health Provider

Parents described personal negative experiences with medications, which may have contributed to their current hesitation around ADHD medication for themselves and for their children. These prior negative experiences with medications appear to have a global effect on medication hesitation, such that the negative experiences were not specific to ADHD medications. For example, parents described experiencing significant side effects with antidepressants and generalized the experience to medications for other conditions, such as ADHD. Providers recounted similar experiences they have heard from the families that they work with and acknowledged that early classes of medications indeed often had severe side effects, which understandably contribute to parents’ continued hesitation regarding medications. Suggestions for discussing ADHD medications included a shared decision-making approach in which providers ask parents about their concerns regarding medication in order to address them directly. Providers also discussed the value of sharing positive anecdotes of medication use, such as “*when you find something that works, [I’ve] seen kids go from failing to getting into Ivy League schools, [gaining] confidence, self-esteem.”*

## Discussion

In the current pilot study, a protocol to screen multiplex ADHD families was implemented in urban pediatric primary care clinics serving predominantly racially minoritized families and families insured through Medicaid. In the first 6 months of implementing the screening protocol, approximately 1 in 5 families attending well-child visits who completed the screeners had elevated parent and child ADHD symptoms. However, overall screening completion rate was low (18.28%). Stakeholder input provided context and offered a deeper understanding of factors that may have contributed to the low ADHD screening completion rates. Themes largely converged between the types of stakeholders, but divergence was also observed between parents and providers/staff. Although the inclusion of multiple types of stakeholders provided important perspectives from an array of individuals who play a role or are impacted by the implementation of multiplex ADHD screening and treatment, we had a small number of participants within each type of stakeholder, limiting generalizability of our findings. As such, current findings represent preliminary data on an understudied topic.

First, although providers in the study expressed enthusiasm for integrating parent mental health with pediatric care, parents expressed significant hesitation about this approach. Furthermore, there is tension between parents’ preference for having conversations with trusted providers about parent mental health and the structural and logistical reality of limited time for pediatric providers to have these sensitive conversations during a well-child visit. On the one hand, the relationship built between providers and parents offers providers the unique opportunity to inquire about more sensitive and potentially uncomfortable topics, such as parent’s own ADHD. This aligns with previous literature identifying Black and/or Latino communities and low-income groups preferring to receive mental health information from a credible, trusted source and someone that is known to them, such as a family doctor, and a preference for receiving mental health information in a familiar setting [39, 40]. Pediatricians within a local community clinic may be particularly suited to provide information about parent ADHD when working with under-resourced families. On the other hand, pediatricians may have limited time to have one-on-one conversations within the context of well-child visits. Although the current sample was small, parent hesitation and the tension between parent preference for conversation and limited provider time raise questions about where screening for parent ADHD may be best situated and what may be the best screening process. For example, the incorporation of family navigators or parent advocates may increase feasibility of mental health screening in underserved communities. Family navigators or parent advocates are peers or paraprofessionals from the community, usually of similar backgrounds and experiences, that help families access and coordinate healthcare services, provide education and help empower families, and address barriers to care [41, 42]. Several strategies have been recommended to incorporate family navigators in pediatric primary care settings to help increase the integration of mental health screening in the domains of informational and educational support, instructional and skill development support, instrumental support, advocacy support, and emotional and affirmational support [43]. Alternative protocols may be to only screen for parent ADHD within pediatric primary care if their children screened positive for ADHD risk (although this approach will inadvertently miss some parents with ADHD), or to screen for parent ADHD in adult primary care for adults who have children (although connecting this information to child systems to improve child functioning remains a challenge), or to screen for parent and child ADHD within a family medicine setting. In addition, strategies to increase parent response rate may include provision of clear rationale on the screener for asking about parent ADHD, presenting screeners in visually engaging and easy to understand formats, leveraging technology to make screeners more appealing (e.g., gamify the questionnaire and include rewards or incentives for completing questions), administering the screeners via patient portals in advance of the visit coupled with reminders for completion, or have providers highlight the importance of completing screeners. Future research can examine and compare these approaches and strategies and seek feedback from parents and providers on what is the most feasible and sustainable approach to identify both parent and child ADHD.

Aside from logistical considerations, stakeholder input also provided insight on how to sensitively broach the topic of parent ADHD within a pediatric primary care setting given the continued inequities in mental healthcare and research with under-resourced families and challenges related to stigma. A key clinical takeaway is to attend to historical and continued oppression and negative experiences of individuals with marginalized identities and how these experiences impact their decision-making around ADHD care for themselves and their child. Significant stigma around mental health persists among communities of color and communities with socioeconomic disadvantages [44, 45]. Parents in this study described feelings of hesitation regarding identifying themselves or their child as having ADHD, with particular emphasis on negative consequences associated with identifying Black children with ADHD (e.g., worries around being treated differently by others and impulse control and behavioral problems that may result in legal consequences). These fears stem from a history of systemic racism, mistreatment, and over policing of communities of color and neighborhoods with high rates of poverty. Research has documented lower quality of care and treatment retention among Black youth with ADHD compared to their White counterparts [12–14], particularly among those with low-income status and/or without health insurance coverage [46, 47]. Concerns about potential consequences of a diagnosis of ADHD highlight the importance of having more in-depth conversations without undermining their stigma-related concerns and yet helping them get the best evidence-based care for themselves and their child. Empathy and awareness of these concerns with ethnic minoritized families and families living in under-resourced settings is likely to increase the families’ willingness to share concerns about mental health [48].

This cultural sensitivity is particularly important if part of the treatment plan for ADHD includes the potential for medication which is associated with more mistrust and stigma. Parents in the study expressed concerns about medication for ADHD for themselves or their children based on past experiences. This finding is consistent with Glasofer et al. [49] describing Black caregivers were less likely to think ADHD medication is efficacious and have concerns about medications as a form of social control on the Black community. Communities with diverse beliefs about the causes and treatment of ADHD may be hesitant to seek help from mental health providers who they perceive as lacking in cultural competence or as holding cultural values incompatible with their own [50]. These findings highlight the importance of exploring beliefs and knowledge about ADHD and ADHD treatment and engaging in shared decision-making. Furthermore, this concern highlights the importance of discussing the benefits and costs of varying treatment options for ADHD and other mental health concerns (e.g., beginning with behavioral interventions or organizational skills training before medications). Additionally, our qualitative findings highlight the importance of continuing research on the impact of parent ADHD on child outcomes and to increase the representation of culturally and economically diverse families in research. To date, the majority of research on child ADHD, parent ADHD, and interventions for multiplex families have been conducted with middle-class White children and families [4, 5].

## Limitations

Current findings must be considered in light of the following. This study represents initial implementation efforts and screening completion and rates of identifying multiplex ADHD families may vary with additional implementation supports and inclusion of a larger sample. Although the involvement of the stakeholder group provided the opportunity to gather perspectives of individuals occupying diverse roles within the pediatric primary care system, the group format precluded us from more in-depth interviews with individuals to fully understand their varying perspectives. Further, having a combined advisory group may have inadvertently created power differentials and impacted participants’ comfort level with sharing within the larger group. The stakeholder group was also limited in its sample representation (e.g., only two to three individuals from each representative category, members were not randomly selected, which limits the racial/ethnic and gender diversity of members), which may limit the generalizability of qualitative themes.

## Conclusions

Like many other psychological disorders, ADHD runs in families and pediatric primary care represents a promising context for routine screening of multiplex ADHD families. Yet, many issues are at play, both attitudinal and structural, when engaging with families living in under-resourced settings due to continued systemic inequities, mistrust, and stigma. The qualitative themes generated from the current pilot study represent a first step in understanding the perspectives and experiences of stakeholders – parents of children receiving care and providers and staff providing care at urban pediatric primary care clinics. This work and future research inspired by it can lead us closer to discovering the best way to identify and treat familial ADHD.

## Supplementary Information

**Additional file 1: Supplemental Table 1.** Washington DC Study Context.

## Data Availability

The datasets used and/or analyzed during the current study are available from the corresponding author on reasonable request.

## Abbreviations

ADHD: Attention-deficit/hyperactivity disorder
SES: Socioeconomic status
CNH: Children’s National Hospital
AAP: American Academy of Pediatrics
AAPI: Asian American/Pacific Islander
SDQ: Strengths and Difficulties Questionnaire
