# Supplementary material for: Screening for parent and child ADHD in urban pediatric primary care: pilot implementation and stakeholder perspectives
Source: BMC Pediatr. 2023 Jul 13;23:354. doi: 10.1186/s12887-023-04082-2 (PMC10339482; doi:10.1186/s12887-023-04082-2)
Supplement: Supplementary file 1 — Additional file 1: Supplemental Table 1. Washington DC Study Context. [file 12887_2023_4082_MOESM1_ESM.docx]

**Supplemental Table 1**

**Washington DC Study Context**

At the time of this study, the screening protocol for parent and child ADHD was implemented at three clinics across Washington, DC: two located in Southeast DC (Ward 8; serving Wards 7 and 8), and one located in Northwest DC (Ward 5). Table 1 displays demographic data from the DC Health Matters Collaborative (https://www.dchealthmatters.org) comparing the service areas of the primary care clinics to all of DC. Overall, the Southeast Wards have predominantly African American/Black residents (94.12%), and the Northwest Ward has a majority population of African American/Black residents (54.53%). Particularly in the Southeast Wards, 15 to 18% of families with children live below the federal poverty threshold, rates much higher than the overall rate of metro DC. Although the median household income among residents in Ward 5 is comparable to the metro DC average, there is notable variability in median household income levels across racial groups: African American/Black residents in Ward 5 had a median household income of $69,873 and White residents in Ward 5 had a median household income of $179,284. The education level of residents in the two Southeast Wards tend to be lower than the metro average (half of the residents’ highest level of education is High School or below). Residents from the Northeast Ward has levels of educational attainment comparable to metro DC. All three neighborhoods also have low levels of opportunity and resources for children’s healthy development across indices of education (e.g., education enrollment, graduate rate, school poverty, teacher experience), health/environment (e.g., access to healthy food, access to green space, industrial pollutants), and social/economic domains (e.g., employment, poverty, homeownership, single-headed households) (www.diversitydatakids.org). For example, the Southeast area where the primary care clinic is located has “Very Low” level of child opportunities and the Northwest area has “Low” level of child opportunities (www.diversitydatakids.org).
